# Synthesis, crystal structure and Hirshfeld surface analysis of bromido­tetra­kis­[5-(prop-2-en-1-yl­sulf­an­yl)-1,3,4-thia­diazol-2-amine-κ*N* ^3^]copper(II) bromide

**DOI:** 10.1107/S2056989024002652

**Published:** 2024-03-26

**Authors:** Aziz Atashov, Mukhlisakhon Azamova, Daminbek Ziyatov, Zamira Uzakbergenova, Batirbay Torambetov, Tamas Holczbauer, Jamshid Ashurov, Shakhnoza Kadirova

**Affiliations:** a National University of Uzbekistan named after Mirzo Ulugbek, 4 University St., Tashkent, 100174, Uzbekistan; b Karakalpak State University, 1 Ch. Abdirov St. Nukus, 230112, Uzbekistan; c Institute of Organic Chemistry, Research Centre for Natural Sciences, 2 Magyar Tudosok Korutja, H-1117 Budapest, Hungary; d Institute of Bioorganic Chemistry, Academy of Sciences of Uzbekistan, M. Ulugbek, St, 83, Tashkent, 100125, Uzbekistan; Universidad de la Repüblica, Uruguay

**Keywords:** crystal structure, copper(II), 1,3,4-thia­diazole, hydrogen bonding, Hirshfeld surface analysis

## Abstract

A novel cationic complex, bromido­tetra­kis­[5-(prop-2-en-1-ylsulfan­yl)-1,3,4-thia­diazol-2-amine-κ*N* ^3^]copper(II) bromide, [CuBr](C_5_H_7_N_3_S_2_)_4_Br, was synthesized. The complex crystallizes with fourfold mol­ecular symmetry in the tetra­gonal space group *P*4/*n*. The Cu^II^ atom exhibits a square-pyramidal coord­ination geometry. The Cu atom is located centrally within the complex, being coordinated by four nitro­gen atoms from four AAT mol­ecules, while a bromine anion is located at the apex of the pyramid. The amino H atoms of AAT inter­act with bromine from the inner and outer spheres, forming a two-dimensional network in the [100] and [010] directions. Hirshfeld surface analysis reveals that 33.7% of the inter­mol­ecular inter­actions are from H⋯H contacts, 21.2% are from S⋯H/H⋯S contacts, 13.4% are from S⋯S contacts and 11.0% are from C⋯H/H⋯C, while other contributions are from Br⋯H/H⋯Br and N⋯H/H⋯N contacts.

## Abstract 2

The mol­ecular and crystal structure of the bromido­tetra­kis­[5-(prop-2-en-1-ylsulfan­yl)-1,3,4-thia­diazol-2-amine-κ*N* ^3^]copper(II) bromide complex was studied and Hirshfeld surfaces and fingerprint plots were generated to investigate the various inter­molecular inter­actions.

## 1. Chemical context

Nitro­gen-containing heterocycles are a promising class of ligands for the synthesis of transition-metal complexes that are strongly responsive to the changes in external conditions (Lavrenova *et al.*, 2023 ▸). Derivatives of 1,3,4-thia­diazole represent a relatively new class of compounds that demonstrate a broad array of biological activities, making them of significant inter­est to various fields in medicinal chemistry and pharmacology worldwide (Gowramma *et al.*, 2018 ▸; Kaviarasan *et al.*, 2020 ▸; Upadhyay & Mishra, 2017 ▸; Yusuf *et al.*, 2008 ▸). 1,3,4-Thia­diazole derivatives exhibit many biological properties, such as anti­microbial (Li *et al.*, 2014 ▸; Chen *et al.*, 2019 ▸), anti­tuberculosis (Foroumadi *et al.*, 2004 ▸; Kolavi *et al.*, 2006 ▸), anti­oxidant (Jakovljević *et al.*, 2017 ▸; Swapna *et al.*, 2013 ▸), anti­cancer (Altıntop *et al.*, 2018 ▸; Aliabadi, 2016 ▸), herbicidal (Wang *et al.*, 2011 ▸) and anti­fungal (Chen *et al.*, 2007 ▸; Karaburun *et al.*, 2018 ▸) activities. In addition, a limited number of studies mention the utilization of diverse thia­diazo­les as ligands in the synthesis of biologically active metal complexes (Huxel *et al.*, 2015 ▸; Chandra *et al.*, 2015 ▸; Hangan *et al.*, 2015 ▸).

The strong complexing capability of thia­diazole derivatives is associated with the existence of numerous sulfur and nitro­gen atoms and the distinctiveness of its structure, specifically, the presence of unshared electron pairs and donor characteristics. They generate complexes with elements whose ions possess partially vacant *d*-orbitals or occupied *d*-orbitals and a low positive charge, exhibiting various polyhedral structures. In this context, investigating the complex-forming properties of thia­diazole derivatives is pertinent in delineating the characteristics of the mol­ecular and electronic structure of the original ligands and the stereochemistry of the coordination polyhedron (Hassan *et al.*, 2018 ▸). This study focuses on the synthesis, examination of the structure, and characteristics of the [Cu(*L*)4Br]Br complex, where *L* is 2-amino-5-allyl­thio-1,3,4-thia­diazole (AAT), employing single-crystal X-ray diffraction (SC-XRD). 
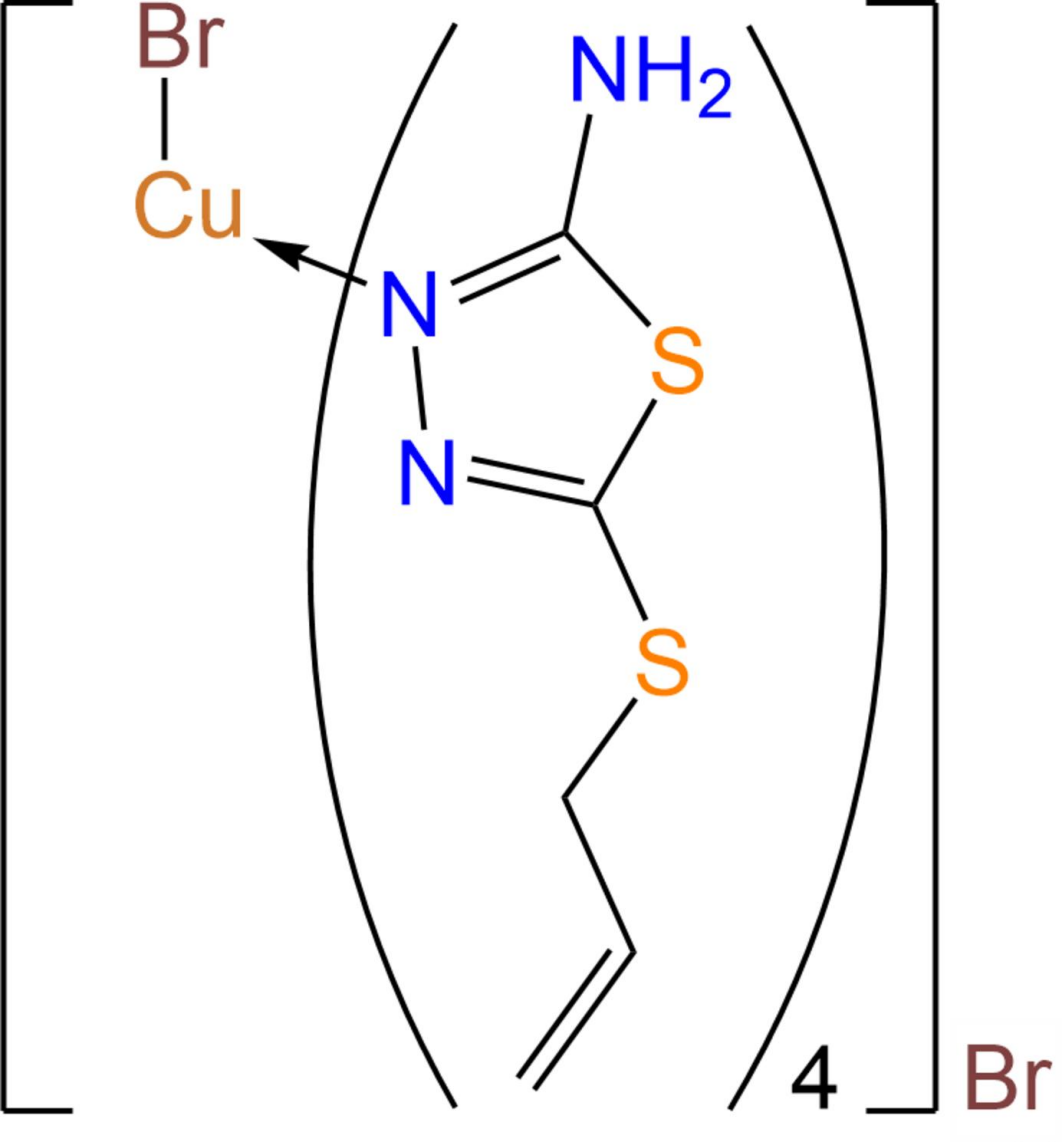


## 2. Structural commentary

**Figure 1:**
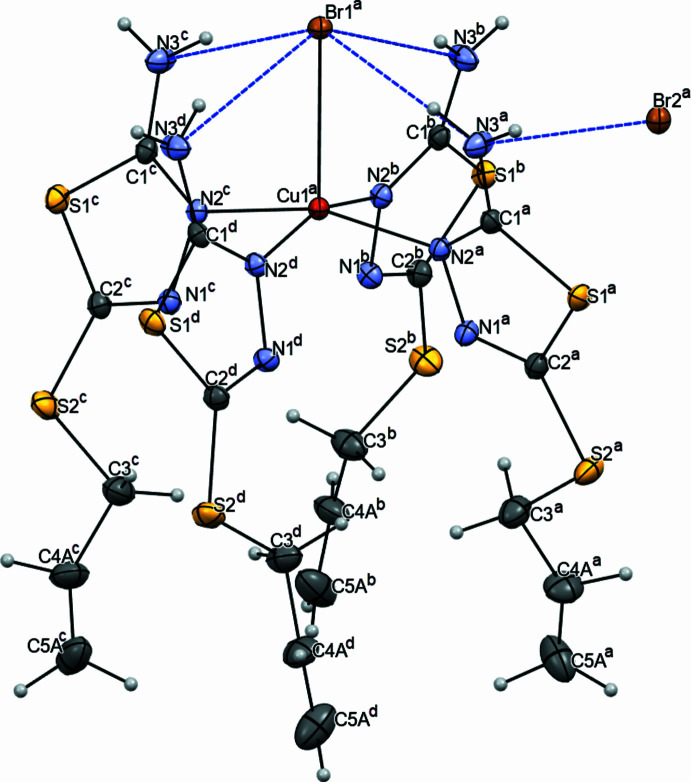
Mol­ecular structure of the complex [Cu(AAT)_4_Br]Br. Displacement ellipsoids are shown with 20% probability level for clarity. Symmetry codes: (a) −*x*, *y*, *z*; (b) − 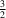 − *y*, *x*, *z*; (c) − 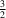 − *x*, 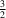 − *y*, *z*; (d) −*y*, 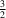 − *x*, *z*.

The crystals of [Cu(AAT)_4_Br]Br possess an ionic-mol­ecular structure. The complex crystallizes in the fourfold tetra­gonal system, space group *P*4/*n*, and the asymmetric unit comprises one mol­ecule of 2-amino-5-allyl­thio-1,3,4-thia­diazole (AAT), one Cu^2+^ ion with a multiplicity of 0.25, and Br^−^ ions in two positions with multiplicities of 0.25 each. The Br^−^ ions occupy special positions on fourfold axes, and this symmetry transformation generates the formula unit. In [Cu(AAT)_4_Br]Br, the copper atom exhibits a square-pyramidal geometry and its coordination sphere includes four nitro­gen atoms (N2) from the heterocyclic ligands and a bromine anion at the top of the pyramid. These nitro­gen atoms lie in one plane. The planar AAT mol­ecules are nearly perpendicular to this plane, exhibiting a slight twist of the Br1CuN2 planes. All the amino groups are in a *syn* arrangement. One of the Br^−^ ions is integrated into the inner coordination sphere, while the second Br- ion resides in the outer sphere (Fig. 1 ▸). As a result, the inner coordination sphere of the complex takes the shape of a tetra­gonal pyramid, where the basal positions are filled by nitro­gen atoms from the 2-amino-5-allyl­thio-1,3,4-thia­diazole ligands, and the apical position is occupied by the Br^−^ ion.

The Cu—Br bond length in the compound measures 2.7474 (7) Å, closely resembling the Cu—Br distance in the [Cu*L*_4_Br_2_](H_2_O)_2_ mol­ecule, which is 2.9383 Å (Berezin *et al.*, 2018 ▸). Apparently, the binding of the Br^−^ ion into the inner coordination sphere induces a distortion of the CuN_4_ plane. The effect of this distortion is to the reduce N—Cu—N coordination angles [88.446 (18) and 161.04 (11)°], in contrast to the angles of 90 and 180° expected in an ideal square-planar structure. The sum of bond angles at the Cu atom is 353.8°. The Cu atom deviates from the (N2)_4_ plane toward Br^−^ by 0.333 Å. The length of the Cu—N coordination bonds is 2.0206 (17) Å, similar to those bonds in analogous complexes. For instance, in nitrato-tetra­kis­(2-amino-5-ethyl-1,3,4-thia­diazole)copper(II) nitrate, the average Cu—N bond length is 2.003 Å (Kadirova *et al.*, 2008 ▸), aligning with the sum of the covalent radii of Cu and N.

Additionally, the Br1 atom participates in the formation of an intra­mol­ecular hydrogen bond with hydrogen atoms of four amino groups NH_2_ simultaneously (Table 1 ▸). By comparing the structures of some complexes based on 2-amino-1,3,4-thia­diazole derivatives, we see that copper(II) bromide, in contrast to chlorides and acetates of cobalt(II) and zinc(II) (Camí *et al.*, 2005 ▸; Song *et al.*, 2012 ▸; Wang *et al.*, 2009 ▸; Kadirova *et al.*, 2008 ▸; Ishankhodzhaeva *et al.*, 2000 ▸, 2001 ▸), exhibits a distinct behavior when reacted with 2-amino-5-all­ylthio-1,3,4-thia­diazole under identical conditions. Instead of forming a tetra­hedral mol­ecular complex as might be anti­cipated from analytical data, copper(II) bromide forms the tetra­gonal–pyramidal cationic complex [Cu(AAT)_4_Br]Br.

## 3. Supra­molecular features

**Figure 2:**
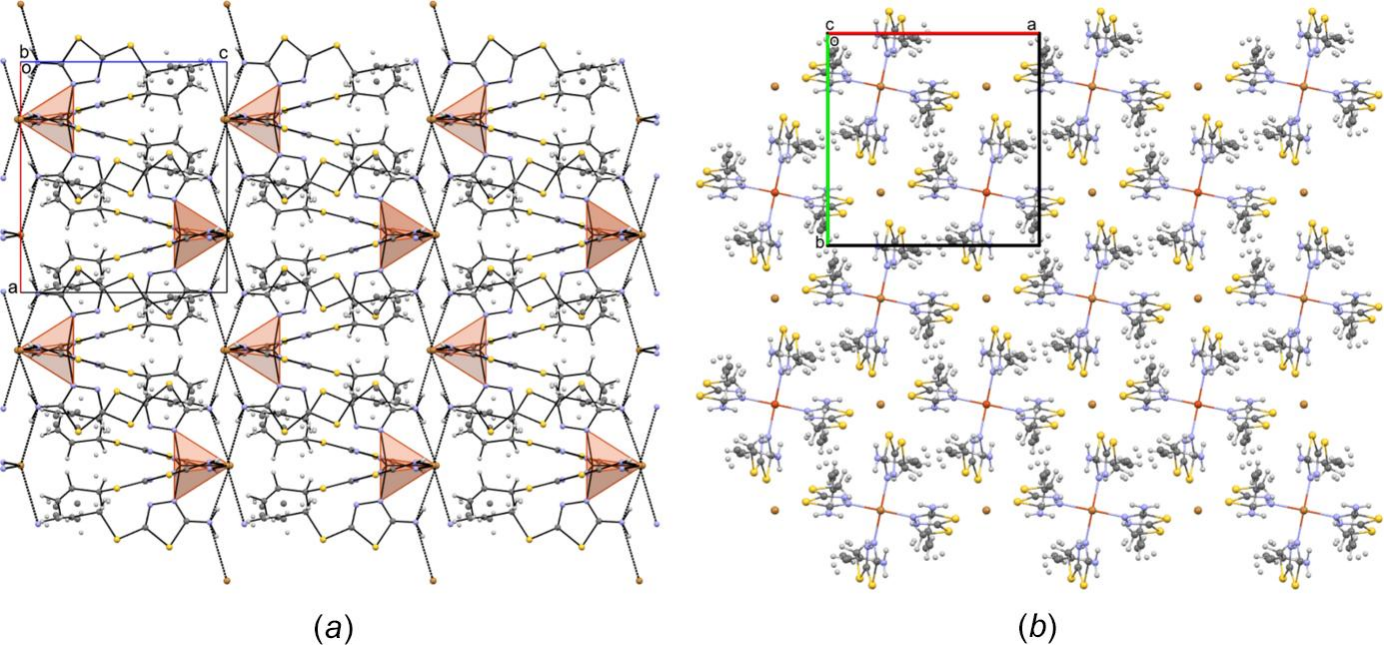
Packing of [Cu(AAT)_4_Br]Br complex mol­ecules in the crystal structure in projections along the (*a*) *b* and (*b*) *c* crystallographic axis. Hydrogen bonds are indicated by blue dashed lines.

In the crystal structure of [Cu(AAT)_4_Br]Br, in addition to the aforementioned intra­molecular hydrogen bonds, there exist inter­mol­ecular hydrogen bonds. The second bromide ion, positioned in the outer sphere, forms a hydrogen bond with the second (not participating in the intra­mol­ecular hydrogen bond) hydrogen atom of the amino group N3H2 (Table 1 ▸). The outer-sphere Br2 ion also resides on the fourfold axis, resulting in the generation of a layer in the crystal perpendicular to the fourfold axis due to this symmetry transformation. As a result, in the crystal packing, the cationic coordination complexes form columns along the [001] crystallographic axis (Fig. 2 ▸). The bromine anions of the outer sphere of the complex are located between the columns due to the formation of the N3—H3*B*⋯Br2 inter­molecular hydrogen bonds with the amino groups of the ligand (Table 1 ▸).

**Figure 3:**
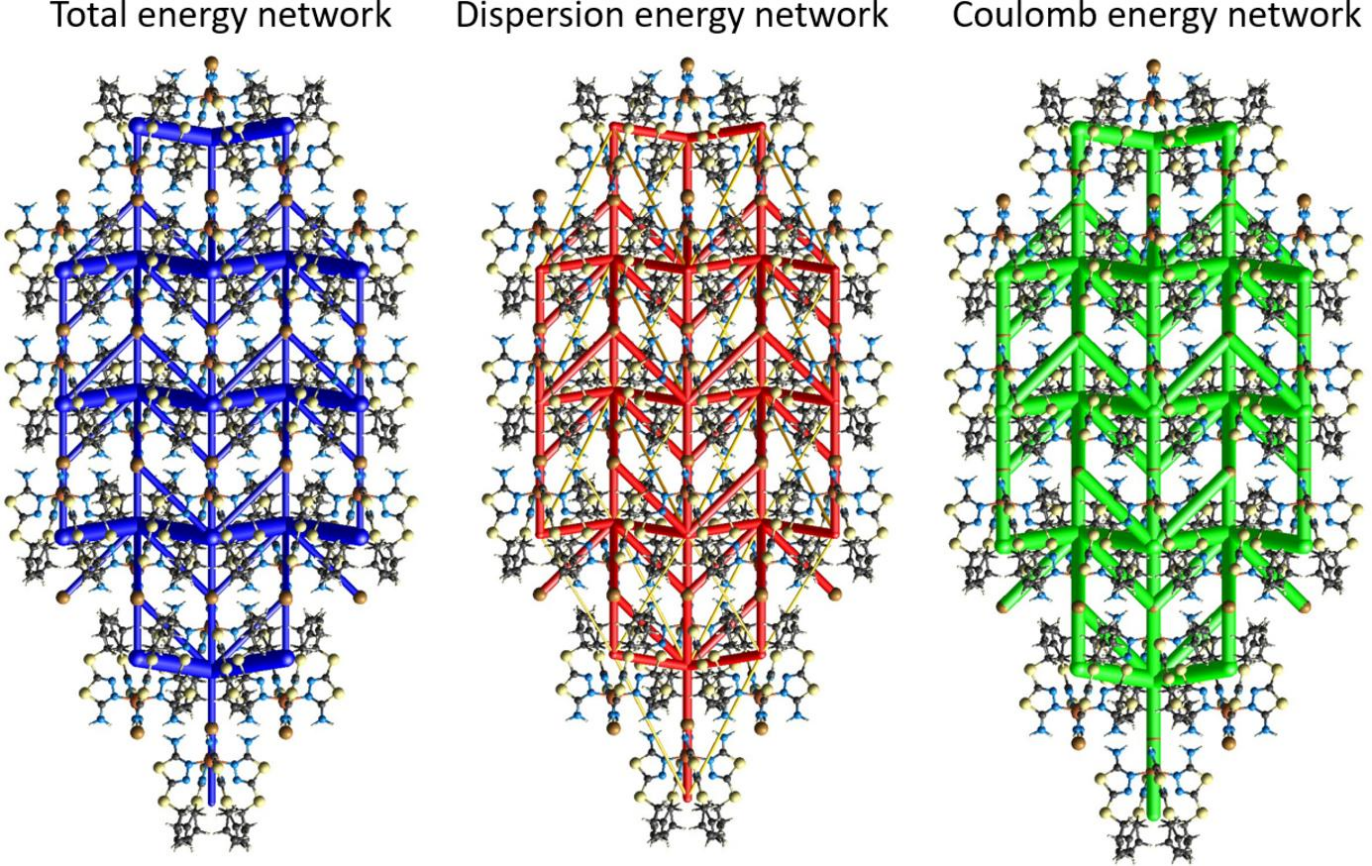
Inter­action energy calculations within the structure were performed using the HF method (HF/3–21 G) (*CrystalExplorer17*; Spackman *et al.*, 2021 ▸. The thickness of the tube represents the value of the energy. The distribution of the inter­actions according to type shows strong inter­actions along the crystallographic *a*-axis direction (the largest values are represented here). The total energy framework (in blue) and its two main components, dispersion (in green) and Coulombic energy (in red), are shown for a cluster around a reference mol­ecule also exhibit stronger inter­actions along the crystallographic *a*-axis direction.

The inter­action energies of the secondary inter­actions system within the structure were calculated using the HF method (HF/3-21G) in *CrystalExplorer17* (Spackman *et al.*, 2021 ▸). Although these calculations may not yield precise values for an ionic inter­action, they effectively highlight the direction of strong inter­actions. The result shows the total energy (*E*_tot_), which is the sum of the Coulombic (*E*_ele_), polar (*E*_pol_), dispersion (*E*_dis_) and repulsive (*E*_rep_) contributions. The four energy components were scaled in the total energy (*E*_tot_ = 1.019*E*_ele_ + 0651*E*_pol_ + 0901*E*_dis_ + 0.811*E*_rep_). The inter­action energies were investigated for a 3.8 Å cluster around the reference mol­ecule. The calculation reveals two stronger inter­actions within the neighbouring mol­ecules. The strongest inter­action total energy (*E*_tot_) is −112.5 kJ mol^−1^ (∼-27 kcal mol^−1^), with the polar (−30.1 kJ mol^−1^), dispersion (−123.3 kJ mol^−1^), Coulombic (−58.5 kJ mol^−1^) and repulsive (96.0 kJ mol^−1^) energies (with green colour) (Fig. 3 ▸).

## 4. Hirshfeld surface analysis

**Figure 4:**
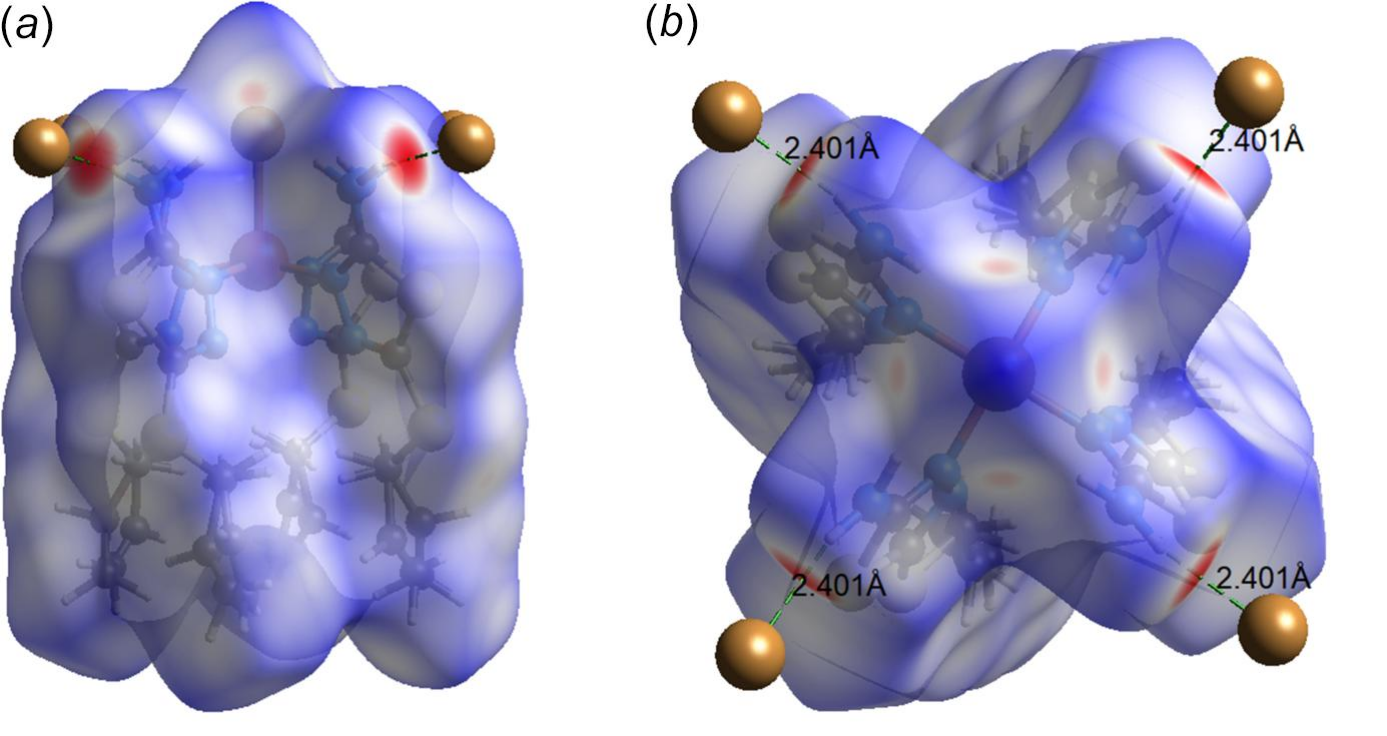
Views of the three-dimensional Hirshfeld surface of the complex [Cu(AAT)_4_Br]^+^ cation plotted over *d*_norm in_ views along the (*a*) [110] and (*b*) [001] directions.

**Figure 5:**
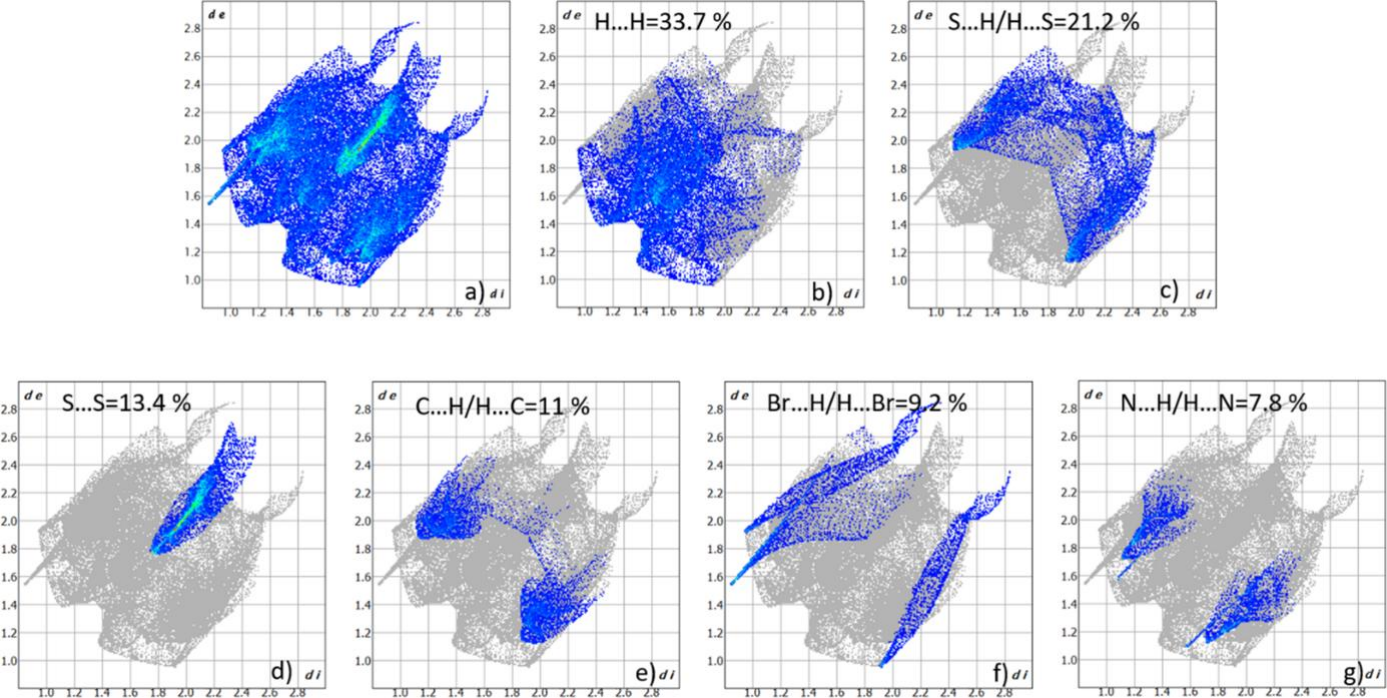
Contributions of the various contacts to the two-dimensional fingerprint plots built using the Hirshfeld surfaces of the title complex.

To further investigate the inter­mol­ecular inter­actions present in the title compound, a Hirshfeld surface analysis was performed, and the two-dimensional (2D) fingerprint plots were generated with *CrystalExplorer17* (Spackman *et al.*, 2021 ▸). Fig. 4 ▸ shows the three-dimensional (3D) Hirshfeld surfaces of the complex with *d*_norm_ (normalized contact distance) plotted. The hydrogen-bond inter­actions given in Table 1 ▸ play a key role in the mol­ecular packing of the complex. The overall 2D fingerprint plot and those delineated into H⋯H, S⋯H/H⋯S, S⋯S, C⋯H/H⋯C, Br⋯H/H⋯Br and N⋯H/H⋯N inter­actions are shown in Fig. 5 ▸. The percentage contributions to the Hirshfeld surfaces from the various inter­atomic contacts are as follows: H⋯H 33.7%, S⋯H/H⋯S 21.2%, S⋯S 13.4, C⋯H/H⋯C 11%, Br⋯H/H⋯Br 9.2% and N⋯H/H⋯N 7.8%. Other minor contributions to the Hirshfeld surface are: S⋯C/C⋯S 1.9% and Br⋯S/S⋯Br 1.6%.

## 5. Database survey

A survey of the Cambridge Structural Database (CSD, version 5.43, update of March 2022; Groom *et al.*, 2016 ▸) revealed that nearly a hundred crystal structures had been reported for complexes of 2-amino-1,3,4-thia­diazole derivatives and a number of metal ions, including Mn, Fe, Co, Ni, Cu, Zn, Mo, Ag, Pd, Cd, Sn, Re, Pt, Au and Hg, twelve of which are for Cu complexes. Six structures exhibit tetra­gonal–pyramidal polyhedra (HONDOG, Torambetov *et al.*, 2019 ▸; RUFQIT, Kadirova *et al.*, 2008 ▸; SUZVOY, SUZVUE, Lynch & Ewington, 2001 ▸; XIGWIU, Camí *et al.*, 2005 ▸; ZEKWOE, Gurbanov, *et al.*, 2018 ▸). In seven structures, AAT is attached to metal ions, making *p*-complexes (ODAPOC, Slyvka *et al.*, 2022 ▸; CEDSEM, Slyvka, 2017*a* ▸; ESIBUG, Slyvka *et al.*, 2021 ▸; HAJLUC, Ardan *et al.*, 2017 ▸; HAJMAJ, HAJMIR, Ardan *et al.*, 2017 ▸; YEBNAX, Slyvka, 2017*b* ▸). However, no complexes of CuBr_2_ based on 2-amino-1,3,4-thia­diazole derivatives have been documented in the CSD.

## 6. Synthesis and crystallization

The ligand 2-amino-5-allyl­thio-1,3,4-thia­diazole (AAT) was synthesized by the method of Toshmurodov *et al.* (2016 ▸), yield: 93%, m.p. = 388–390 K. CuBr_2_·4H_2_O (0.296g, 1 mmol) was added under continuous stirring to a solution of AAT (0.692 g, 4 mmol) dissolved in 10 ml of methanol. The resulting dark-green solution was stirred for 3 h and was then left to stand at room temperature. After one week, green crystals suitable for X-ray diffraction were obtained (yield 86%) by the slow evaporation of the solvent, m.p. = 458–460 K.

## 7. Refinement

Crystal data, data collection and structure refinement details are summarized in Table 2 ▸. H atoms were positioned geometrically (N—H = 0.86 Å, C—H = 0.83–0.97 Å) and refined as riding with *U*_iso_(H) = 1.2*U*_eq_(C, N).

## Supplementary Material

Crystal structure: contains datablock(s) I. DOI: 10.1107/S2056989024002652/ny2003sup1.cif

Structure factors: contains datablock(s) I. DOI: 10.1107/S2056989024002652/ny2003Isup2.hkl

CCDC reference: 2341909

Additional supporting information:  crystallographic information; 3D view; checkCIF report

## Figures and Tables

**Table 1:** Hydrogen-bond geometry (Å, °)

*D*—H⋯*A*	*D*—H	H⋯*A*	*D*⋯*A*	*D*—H⋯*A*
N3—H3*A*⋯Br1	0.86	2.52	3.3265 (1)	157
N3—H3*B*⋯Br2	0.86	2.54	3.3685 (1)	162
C5*A*—H5*AA*⋯Br1^i^	0.93	3.03	3.95 (2)	175

Symmetry code: (i) 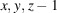 .

**Table 2:** Experimental details

Crystal data	
Chemical formula	[CuBr(C_5_H_7_N_3_S_2_)_4_]Br
*M*_r_	916.38
Crystal system, space group	Tetragonal, *P*4/*n*
Temperature (K)	293
*a*, *c* (Å)	12.69368 (9), 11.35879 (13)
*V* (Å^3^)	1830.24 (3)
*Z*	2
Radiation type	Cu *K*α
μ (mm^−1^)	7.95
Crystal size (mm)	0.12 × 0.08 × 0.03

Data collection	
Diffractometer	XtaLAB Synergy, Single source at home/near, HyPix3000
Absorption correction	Multi-scan (*CrysAlis PRO*; Rigaku OD, 2020 ▸)
*T*_min_, *T*_max_	0.626, 1.000
No. of measured, independent and observed [*I* > 2σ(*I*)] reflections	18450, 1746, 1597
*R*_int_	0.036
(sin θ/λ)_max_ (Å^−1^)	0.609

Refinement	
*R*[*F* ^2^ > 2σ(*F* ^2^)], *wR*(*F* ^2^), *S*	0.025, 0.065, 1.05
No. of reflections	1746
No. of parameters	118
H-atom treatment	H-atom parameters constrained
Δρ_max_, Δρ_min_ (e Å^−3^)	0.26, −0.28

Computer programs: *CrysAlis PRO* (Rigaku OD, 2020 ▸), *SHELXT* (Sheldrick, 2015*a* ▸), *SHELXL2018/3* (Sheldrick, 2015*b* ▸), *OLEX2* (Dolomanov *et al.*, 2009 ▸).

## supplementary crystallographic information

### Crystal data

**Table:**

[CuBr(C_5_H_7_N_3_S_2_)_4_]Br	*D*_x_ = 1.663 Mg m^−^^3^
*M**_r_* = 916.38	Cu *K*α radiation, λ = 1.54184 Å
Tetragonal, *P*4/*n*	Cell parameters from 9121 reflections
*a* = 12.69368 (9) Å	θ = 3.5–70.8°
*c* = 11.35879 (13) Å	µ = 7.95 mm^−^^1^
*V* = 1830.24 (3) Å^3^	*T* = 293 K
*Z* = 2	Block, green
*F*(000) = 918	0.12 × 0.08 × 0.03 mm

### Data collection

**Table:**

XtaLAB Synergy, Single source at home/near, HyPix3000 diffractometer	1597 reflections with *I* > 2σ(*I*)
Detector resolution: 10.0000 pixels mm^-1^	*R*_int_ = 0.036
ω scans	θ_max_ = 69.9°, θ_min_ = 3.9°
Absorption correction: multi-scan (CrysAlisPro; Rigaku OD, 2020)	*h* = −15→15
*T*_min_ = 0.626, *T*_max_ = 1.000	*k* = −11→15
18450 measured reflections	*l* = −13→13
1746 independent reflections	

### Refinement

**Table:**

Refinement on *F*^2^	Hydrogen site location: inferred from neighbouring sites
Least-squares matrix: full	H-atom parameters constrained
*R*[*F*^2^ > 2σ(*F*^2^)] = 0.025	*w* = 1/[σ^2^(*F*_o_^2^) + (0.0273*P*)^2^ + 0.9257*P*] where *P* = (*F*_o_^2^ + 2*F*_c_^2^)/3
*wR*(*F*^2^) = 0.065	(Δ/σ)_max_ < 0.001
*S* = 1.05	Δρ_max_ = 0.26 e Å^−^^3^
1746 reflections	Δρ_min_ = −0.28 e Å^−^^3^
118 parameters	Extinction correction: SHELXL-2016/6 (Sheldrick 2015b), Fc^*^=kFc[1+0.001xFc^2^λ^3^/sin(2θ)]^-1/4^
0 restraints	Extinction coefficient: 0.00018 (7)

### Special details

**Table:**

Geometry. All esds (except the esd in the dihedral angle between two l.s. planes) are estimated using the full covariance matrix. The cell esds are taken into account individually in the estimation of esds in distances, angles and torsion angles; correlations between esds in cell parameters are only used when they are defined by crystal symmetry. An approximate (isotropic) treatment of cell esds is used for estimating esds involving l.s. planes.

### Fractional atomic coordinates and isotropic or equivalent isotropic displacement parameters (Å^2^)

**Table:**

	*x*	*y*	*z*	*U*_iso_*/*U*_eq_	Occ. (<1)
Br1	0.750000	0.750000	1.01564 (4)	0.04943 (15)	
Br2	0.250000	0.750000	1.000000	0.05616 (16)	
Cu1	0.750000	0.750000	0.77377 (5)	0.03890 (16)	
S1	0.39660 (5)	0.70986 (6)	0.72047 (6)	0.06176 (19)	
S2	0.42895 (6)	0.65094 (8)	0.46657 (7)	0.0831 (3)	
N2	0.59625 (14)	0.71817 (14)	0.74447 (16)	0.0470 (4)	
N1	0.57992 (14)	0.68882 (16)	0.62785 (18)	0.0544 (5)	
N3	0.50405 (16)	0.7637 (2)	0.9157 (2)	0.0739 (7)	
H3A	0.560992	0.775718	0.954484	0.089*	
H3B	0.443808	0.771729	0.949189	0.089*	
C1	0.50915 (16)	0.73279 (18)	0.8039 (2)	0.0500 (5)	
C2	0.48136 (19)	0.6828 (2)	0.6037 (2)	0.0574 (6)	
C3	0.5494 (3)	0.6360 (3)	0.3816 (3)	0.0984 (11)	
H3BC	0.580640	0.704195	0.365145	0.118*	0.5
H3BD	0.600153	0.593336	0.423967	0.118*	0.5
H3AA	0.600532	0.688177	0.406669	0.118*	0.5
H3AB	0.579095	0.566715	0.395347	0.118*	0.5
C5A	0.5337 (16)	0.5753 (12)	0.1730 (17)	0.143 (7)	0.5
H5AA	0.587593	0.612982	0.136912	0.171*	0.5
H5AB	0.492013	0.529830	0.128742	0.171*	0.5
C4	0.5259 (9)	0.6502 (7)	0.2475 (9)	0.104 (3)	0.5
H4	0.516751	0.720330	0.226229	0.125*	0.5
C4A	0.5181 (8)	0.5852 (11)	0.2749 (8)	0.112 (3)	0.5
H4A	0.461720	0.541488	0.293578	0.134*	0.5
C5	0.5187 (14)	0.6021 (18)	0.1813 (12)	0.154 (8)	0.5
H5A	0.526312	0.529967	0.192763	0.185*	0.5
H5B	0.504113	0.628295	0.106619	0.185*	0.5

### Atomic displacement parameters (Å^2^)

**Table:**

	*U*^11^	*U*^22^	*U*^33^	*U*^12^	*U*^13^	*U*^23^
Br1	0.05153 (19)	0.05153 (19)	0.0452 (3)	0.000	0.000	0.000
Br2	0.04993 (19)	0.04993 (19)	0.0686 (3)	0.000	0.000	0.000
Cu1	0.0362 (2)	0.0362 (2)	0.0443 (3)	0.000	0.000	0.000
S1	0.0387 (3)	0.0791 (4)	0.0675 (4)	0.0015 (3)	−0.0065 (3)	0.0060 (3)
S2	0.0637 (4)	0.1138 (7)	0.0718 (5)	−0.0029 (4)	−0.0179 (4)	−0.0189 (4)
N2	0.0405 (9)	0.0489 (10)	0.0517 (10)	−0.0013 (8)	−0.0029 (8)	0.0008 (8)
N1	0.0450 (10)	0.0610 (12)	0.0573 (11)	−0.0041 (9)	−0.0020 (8)	−0.0070 (9)
N3	0.0430 (11)	0.121 (2)	0.0573 (13)	0.0096 (12)	0.0002 (9)	−0.0060 (13)
C1	0.0388 (11)	0.0538 (12)	0.0574 (13)	0.0016 (9)	−0.0018 (9)	0.0079 (10)
C2	0.0489 (13)	0.0608 (14)	0.0626 (15)	−0.0020 (11)	−0.0058 (11)	−0.0008 (11)
C3	0.084 (2)	0.131 (3)	0.080 (2)	−0.004 (2)	−0.0020 (18)	−0.023 (2)
C5A	0.168 (13)	0.095 (7)	0.165 (16)	−0.031 (7)	0.075 (10)	−0.020 (7)
C4	0.173 (9)	0.068 (5)	0.070 (6)	−0.034 (5)	0.017 (5)	0.007 (4)
C4A	0.117 (7)	0.141 (10)	0.077 (6)	0.013 (7)	0.024 (5)	−0.019 (6)
C5	0.121 (9)	0.30 (2)	0.038 (4)	0.016 (12)	−0.010 (5)	−0.005 (8)

### Geometric parameters (Å, º)

**Table:**

Br1—Cu1	2.7474 (7)	C3—H3BC	0.9700
Cu1—N2^i^	2.0206 (17)	C3—H3BD	0.9700
Cu1—N2	2.0206 (17)	C3—H3AA	0.9700
Cu1—N2^ii^	2.0206 (17)	C3—H3AB	0.9700
Cu1—N2^iii^	2.0206 (17)	C3—C4	1.562 (11)
S1—C1	1.739 (2)	C3—C4A	1.429 (10)
S1—C2	1.742 (3)	C5A—H5AA	0.9300
S2—C2	1.741 (3)	C5A—H5AB	0.9300
S2—C3	1.818 (4)	C5A—C4A	1.181 (19)
N2—N1	1.392 (3)	C4—H4	0.9300
N2—C1	1.308 (3)	C4—C5	0.973 (18)
N1—C2	1.283 (3)	C4A—H4A	0.9300
N3—H3A	0.8600	C5—H5A	0.9300
N3—H3B	0.8600	C5—H5B	0.9300
N3—C1	1.331 (3)		
			
N2^i^—Cu1—Br1	99.48 (5)	S2—C3—H3BC	110.7
N2^iii^—Cu1—Br1	99.48 (5)	S2—C3—H3BD	110.7
N2^ii^—Cu1—Br1	99.48 (5)	S2—C3—H3AA	109.6
N2—Cu1—Br1	99.48 (5)	S2—C3—H3AB	109.6
N2^ii^—Cu1—N2^iii^	88.446 (18)	H3BC—C3—H3BD	108.8
N2^ii^—Cu1—N2	88.446 (18)	H3AA—C3—H3AB	108.1
N2^iii^—Cu1—N2^i^	88.446 (18)	C4—C3—S2	110.2 (5)
N2^i^—Cu1—N2	88.445 (18)	C4—C3—H3AA	109.6
N2^ii^—Cu1—N2^i^	161.04 (11)	C4—C3—H3AB	109.6
N2^iii^—Cu1—N2	161.04 (11)	C4A—C3—S2	105.3 (5)
C1—S1—C2	86.58 (11)	C4A—C3—H3BC	110.7
C2—S2—C3	100.25 (14)	C4A—C3—H3BD	110.7
N1—N2—Cu1	110.74 (13)	H5AA—C5A—H5AB	120.0
C1—N2—Cu1	134.66 (16)	C4A—C5A—H5AA	120.0
C1—N2—N1	113.76 (18)	C4A—C5A—H5AB	120.0
C2—N1—N2	111.4 (2)	C3—C4—H4	112.8
H3A—N3—H3B	120.0	C5—C4—C3	134.4 (15)
C1—N3—H3A	120.0	C5—C4—H4	112.8
C1—N3—H3B	120.0	C3—C4A—H4A	106.8
N2—C1—S1	112.94 (18)	C5A—C4A—C3	146.4 (13)
N2—C1—N3	125.1 (2)	C5A—C4A—H4A	106.8
N3—C1—S1	121.96 (17)	C4—C5—H5A	120.0
S2—C2—S1	119.40 (14)	C4—C5—H5B	120.0
N1—C2—S1	115.33 (19)	H5A—C5—H5B	120.0
N1—C2—S2	125.3 (2)		
			
Cu1—N2—N1—C2	−170.89 (17)	C1—S1—C2—S2	−178.55 (17)
Cu1—N2—C1—S1	168.95 (12)	C1—S1—C2—N1	1.2 (2)
Cu1—N2—C1—N3	−9.9 (4)	C1—N2—N1—C2	0.2 (3)
S2—C3—C4—C5	−103 (2)	C2—S1—C1—N2	−1.01 (18)
S2—C3—C4A—C5A	148 (2)	C2—S1—C1—N3	177.9 (2)
N2—N1—C2—S1	−1.0 (3)	C2—S2—C3—C4	−158.1 (4)
N2—N1—C2—S2	178.68 (17)	C2—S2—C3—C4A	166.4 (6)
N1—N2—C1—S1	0.7 (2)	C3—S2—C2—S1	178.11 (19)
N1—N2—C1—N3	−178.2 (2)	C3—S2—C2—N1	−1.6 (3)

Symmetry codes: (i) *y*, −*x*+3/2, *z*; (ii) −*y*+3/2, *x*, *z*; (iii) −*x*+3/2, −*y*+3/2, *z*.

### Hydrogen-bond geometry (Å, º)

**Table:**

*D*—H···*A*	*D*—H	H···*A*	*D*···*A*	*D*—H···*A*
N3—H3*A*···Br1	0.86	2.52	3.3265 (1)	157
N3—H3*B*···Br2	0.86	2.54	3.3685 (1)	162
C5*A*—H5*AA*···Br1^iv^	0.93	3.03	3.95 (2)	175

Symmetry code: (iv) *x*, *y*, *z*−1.
